# Consequences of the Last Glacial Period on the Genetic Diversity of Southeast Asians

**DOI:** 10.3390/genes13020384

**Published:** 2022-02-21

**Authors:** Catarina Branco, Marina Kanellou, Antonio González-Martín, Miguel Arenas

**Affiliations:** 1 Centro de Investigaciones Biomédicas (CINBIO), University of Vigo, 36310 Vigo, Spain; caraujo@uvigo.es (C.B.); mkanellou@bio.auth.gr (M.K.); 2 Department of Biochemistry, Genetics and Immunology, University of Vigo, 36310 Vigo, Spain; 3 School of Biology, Aristotle University of Thessaloniki, 54124 Thessaloniki, Greece; 4 Department of Biodiversity, Ecology and Evolution, University Complutense of Madrid, 28040 Madrid, Spain; antonio@bio.ucm.es

**Keywords:** modern human evolution, population genetics, last glacial period, long-distance dispersal, Southeast Asians

## Abstract

The last glacial period (LGP) promoted a loss of genetic diversity in Paleolithic populations of modern humans from diverse regions of the world by range contractions and habitat fragmentation. However, this period also provided some currently submersed lands, such as the Sunda shelf in Southeast Asia (SEA), that could have favored the expansion of our species. Concerning the latter, still little is known about the influence of the lowering sea level on the genetic diversity of current SEA populations. Here, we applied approximate Bayesian computation, based on extensive spatially explicit computer simulations, to evaluate the fitting of mtDNA data from diverse SEA populations with alternative evolutionary scenarios that consider and ignore the LGP and migration through long-distance dispersal (LDD). We found that both the LGP and migration through LDD should be taken into consideration to explain the currently observed genetic diversity in these populations and supported a rapid expansion of first populations throughout SEA. We also found that temporarily available lands caused by the low sea level of the LGP provided additional resources and migration corridors that favored genetic diversity. We conclude that migration through LDD and temporarily available lands during the LGP should be considered to properly understand and model the first expansions of modern humans.

## 1. Introduction

**Figure 1:**
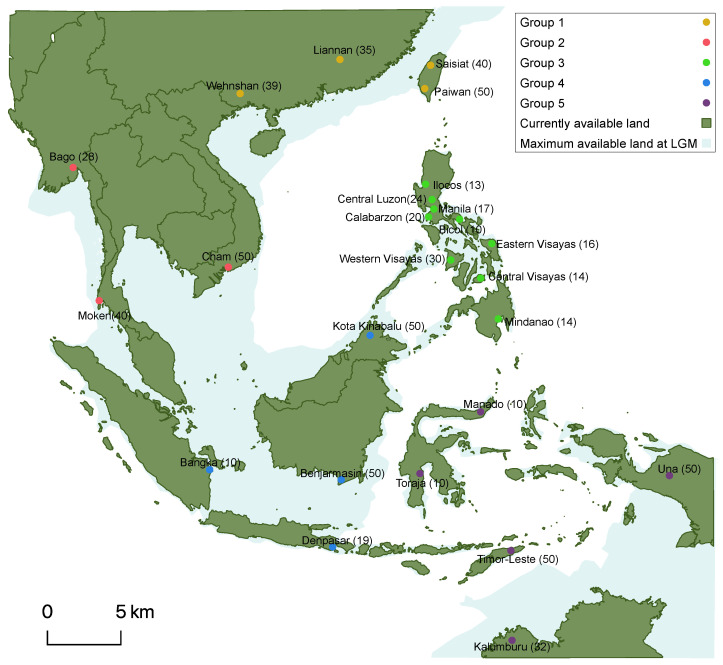
Studied sample locations and land distribution at the present and during the last glacial maximum (LGM) in Southeast Asia. The map shows the sampled populations (for every population the number of individuals is included in parenthesis) and their classification into five geographic groups (shown with colors; for further details, see Table S1). The land available at present is shown in dark green while the land available when the sea level was 120 m below present level (occurring at the LGM) is shown in clear blue.

Despite intense efforts for understanding the evolutionary history of early modern humans (EMHs), diverse past evolutionary events are still unclear. One of them involves the genetic consequences of environmental conditions that occurred during the first expansion of our species. In this regard, the last glacial period (LGP; 125,000–11,000 years ago (ya)) produced large ice sheets in the northern hemisphere regions and increased the extension of deserts [1], forcing humans (as well as other species) to migrate toward more suitable regions and reduce their genetic diversity [2]. These range contractions and range shifts, usually by reducing and moving their living ranges toward lower latitude regions, reduced the genetic diversity in the populations [3,4]. In addition, the LGP overall reduced the sea level about 50 m below present level (BPL), reaching a minimum level (approximately 120 m BPL) during the last glacial maximum (LGM; 25,000–18,000 ya). This low sea level exposed previously submersed lands [1,5] that could be occupied by humans. In summary, the LGP produced a negative impact on human genetic diversity through range contractions [2,3] and habitat fragmentation [6,7], but also, it could be positive for some populations that acquired new habitable lands due to the lower sea level. While the negative influences of the LGP have been widely explored [2,3,4,8,9], little is known about the possible positive effects. Motivated by this question, here we explored the influence of the sea level variation caused by the LGP on the currently observed genetic diversity of modern humans. In order to evaluate it, we selected the region of Southeast Asia (SEA) because it was colonized in the first expansions of EMHs (more than 50,000 ya, during the LGP [10,11]) and it was the basis of the first range expansions toward Oceania [12]. Moreover, SEA is particularly interesting because during the LGP the Asian landmass extended over the Sunda shelf connecting remote islands by land corridors [13] (Figure 1). Therefore, the lower sea level caused by the LGP could have benefited EMHs from SEA not only by providing new lands with resources but also by favoring migration throughout the region [14]. Still, since the sea level lowstand could not connect all the islands of SEA (see Figure 1) [15], maritime migrations using rafts and boats probably took place [12,13]. This is also supported by some studies that detected long-distance dispersal (LDD) events in the colonization of some regions of Asia by EMHs [16,17]. After the LGM (around 18,000 ya), the Sunda shelf flooded (marine transgression) [18,19] and this could have induced a range contraction toward inland regions [20] and a population decline [21].

In order to understand the consequences of the LGP (and LDD) on the genetic diversity of current SEA populations, here we evaluated the fitting of four alternative evolutionary scenarios, which either consider or ignore the LGP (sea level changes) and LDD, with observed genetic data from a variety of SEA populations. We applied approximate Bayesian computation (ABC), based on extensive spatially explicit computer simulations of demographic and genetic data, to identify the best-fitting evolutionary scenario. Next, for the selected scenario, we estimated diverse population genetics parameters (i.e., carrying capacity and migration rates in permanent and temporarily available lands, population size at the onset of the expansion and population growth rate, among others) to understand their role on the observed genetic diversity.

## 2. Material and Methods

In this section, we describe the studied genetic data, the methodology to perform the phylogenetic tree reconstruction and the ABC methods applied to identify the best-fitting evolutionary scenario (considering or ignoring the LGP and LDD) and to estimate the population genetic and demographic parameters.

### 2.1. Genetic Data and Phylogenetic Analysis

The observed genetic dataset (real data) comprised sequences of the mtDNA hypervariable I region (HVR-I, which is the only genetic marker shared by all the SEA populations and it was shown informative to infer other past evolutionary processes [17,22]) obtained from 720 individuals belonging to 25 populations of SEA, where each population is represented by at least 10 individuals (Figure 1 and Table S1; Supplementary Material). The sequences are available from published studies [17,23,24,25,26,27,28,29,30,31,32,33] and following Arenas et al. [17], we only considered sequences belonging to haplogroups older than 20,000 years [34] to avoid lineages originated after the first range expansions of EMHs in SEA. For some analyses (details later), we classified the 25 populations in 5 geographic groups (Figure 1 and Table S1): Group 1 included populations from southeast China and Taiwan; Group 2 included populations from south mainland (Myanmar, Thailand and Vietnam); Group 3 included populations from the Philippine archipelago; and Groups 4 and 5 included the remaining populations located to the north (Borneo, Sumatra and Bali) and south (Sulawesi, Papua, Timor-Leste and north Australia) of the Wallace line, respectively. The sequences were aligned with MAFFT [35].

In order to explore the genetic relationships among the studied samples, we performed a traditional phylogenetic tree reconstruction. We used jModelTest2 [36] to identify the best-fitting substitution model of DNA evolution for the studied data. The selected model, based on the Bayesian Information Criterion (BIC) following Luo et al. [37], was the HKY model [38], with the proportion of invariable sites and rate variation among sites according to a *γ* distribution (HKY +I +G). Next, we inferred a maximum likelihood (ML) phylogenetic tree with RAxML-NG [39] under the previously selected substitution model.

### 2.2. Selection of the Best-Fitting Evolutionary Scenario and Parameters Estimation with Approximate Bayesian Computation

#### 2.2.1. Evolutionary Scenarios and Spatially Explicit Computer Simulations

We evaluated the fitting of four alternative evolutionary scenarios (in terms of paleogeography and paleodemography) with the observed data using ABC. These scenarios were based on two criteria, considering or ignoring the LGP (concerning sea level variation), and considering or ignoring a fraction of migration events presenting LDD: (i) Ignoring the LGP and considering gradual migration (hereafter, *NONE*); (ii) considering the LGP and considering gradual migration (hereafter, *LGP*); (iii) ignoring the LGP and considering gradual migration together with LDD (hereafter, *LDD*); and (iv) considering the LGP and considering gradual migration together with LDD (hereafter, *LGP&LDD)*. Illustrative examples of spatially explicit simulations performed under these evolutionary scenarios are shown in Figures S1–S4 (Supplementary Material).

The spatially explicit computer simulations were performed with the evolutionary framework SPLATCHE3 [40]. Conveniently, this framework implements landscape variation over time (further details about SPLATCHE3 are included in the Supplementary Material), allowing the modeling of the sea level variation with lands that can be habitable only during certain periods of time. The parameters specified to perform the simulations were drawn from uniform prior distributions based on previous studies (Table S2; Supplementary Material). In particular, for all the studied evolutionary scenarios, we simulated a range expansion upon SEA that started at a time sampled from a uniform prior distribution between 60,000 and 70,000 ya (*T*) from current Bangladesh (Figures S1–S4). The effective population size at the onset of the expansion (*N*) varied according to a uniform prior distribution between 25,000 and 75,000 individuals, which is large enough to ensure estimates falling within the range of the prior distribution [41,42]. The population growth rate (*r*) was sampled from a uniform prior distribution ranging between 0.4 and 1.0 and the prior distribution of the migration rate (involving migration of individuals out of a deme, *m*) varied between 0.2 and 0.3. The carrying capacity (*K*) was modeled with a uniform prior between 1000 and 4000 (which in the center of the distribution includes a density of 3 individuals/km^2^ of hunter–gatherers [43]). Evolutionary scenarios considering sea level variation (*LGP* and *LGP&LDD*) included spatial and temporal variation in the carrying capacity and migration rate. In particular, the carrying capacity and migration rate of submersed demes were set to 0, but when previously submersed demes are exposed (due to sea level decrease), their migration rate (*m_temp*) and carrying capacity (*K_temp*) were treated as additional parameters (their values can differ from those for *m* and *K*) and we estimated them separately to assess the impact of the temporary lands on the expansion and genetic diversity of SEA populations. The geographic area of temporarily available lands was based on previous studies [5,15,19,44,45] (Figure 1). In particular, when first populations started to expand along SEA, the sea level was approximately 50 m BPL (Figures S2 and S4). Later, at the beginning of the LGM (25,000 ya), the sea level decreased to 75 m BPL, reaching 100 m BPL at 23,000 ya. The lowest sea level was approximately 120 m BPL at 21,000 ya, when the Sunda shelf and other land connections were fully exposed (Figure 1). Despite the end of the LGM, considered to be around 18,000 ya, the sea level started to increase in some meters only at around 15,000 ya [5,19] and thus we modeled the initial flooding of the temporarily available land regions at that time (Figure S2B). We simulated a total of eight postglacial sea level variations until 7000 ya, when the sea reached its current level [19]. From 15,000 to 13,000 ya, we modeled a sea level increase from 100 to 75 m BPL and, following previous studies [19,44], we modeled a 10 m increase in sea level each 1000 years until 7000 ya (Figure S2B). Regarding the scenarios accounting for LDD, 1% to 5% of migrants were allowed to move by LDD toward any deme located at a maximum distance of 20 demes (500 km), in agreement with Arenas et al. [17] and under the LDD model implemented in SPLATCHE3 [46]. Concerning the modeling of molecular evolution, we applied a mutation rate (*μ*) sampled from a uniform prior distribution (Table S2) that was built considering previous studies on mtDNA evolution in humans [17,47]. Each simulated dataset included a total of 720 genetic sequences belonging to 25 SEA populations (following the observed data). Further details about the parameterization of the spatially explicit computer simulations are provided in the Supplementary Material. For each evolutionary scenario, we performed a total of 300,000 simulations (hence, a total of 1,200,000 simulations considering the four evolutionary scenarios) under the specified prior distributions.

#### 2.2.2. Summary Statistics

Summary statistics (SS) from observed and simulated data were computed with Arlequin ver 3.5.2.2 [48]. The applied SS (described below and in Table S3; Supplementary Material) were designed to consider the relationships between geography and genetic diversity and, conveniently, to distinguish between the studied evolutionary scenarios (Figure S5; Supplementary Material). In total, we selected 11 SS that include (i) genetic differentiation (based on F_ST_) between the three populations located at the northwesternmost region of SEA (Bago, Wehnshan and Liannan) and the three populations located at the southeasternmost region of SEA (Timor-Leste, Una and Kalumburu); (ii) decay in the genetic differentiation (based on F_ST_) between the Bago population (the closest population to the origin of the range expansion) and all the other populations with the geographic distance between them; and (iii) the decay in nucleotide diversity (based on pairwise differences, π) per population with the geographic distance from the origin of the range expansion. The last two SS include the slope of the fitted line obtained from the linear regression between the previously indicated geographic distances and genetic statistics.

#### 2.2.3. Selection among Alternative Evolutionary Scenarios with Approximate Bayesian Computation

We selected the best-fitting evolutionary scenario with the multinomial logistic regression (*mnlogistic*) and the neural networks (*neuralnet*) methods implemented in the *abc* library of R [49]. In order to evaluate the power of these methods for selecting among the studied evolutionary scenarios, we performed a leave-one-out cross-validation based on 100 pseudo-observed simulations (*cv4postpr* function of the *abc* library) and considering a tolerance of 1% [49]. In addition, we evaluated the goodness-of-fit of the SS from the data simulated under every studied evolutionary scenario with the SS of the observed data (distance between the distribution of simulated SS and the observed SS) using principal component analyses [49]. Next, we estimated the posterior probability of each evolutionary scenario fitting the observed data (*postpr* function of the *abc* library [49]) using the *mnlogistic* and *neuralnet* methods and retaining 1% of the simulations (from the total of 300,000 simulations) with SS closer to the SS of the observed data.

#### 2.2.4. Estimation of Evolutionary Parameters with ABC

We estimated the evolutionary parameters of the *LGP&LDD* evolutionary scenario, which was the evolutionary scenario that best fitted the observed data. The parameters estimation was based on the multiple linear regression method implemented in ABCtoolbox [50]. Following previous studies [17,51], we evaluated the robustness of the method in estimating the parameters of the selected evolutionary scenario by the analysis of 100 independent genetic simulations (pseudo-observed data). In particular, we estimated the parameters from the pseudo-observed data retaining 1000 from the total number of simulations (300,000; selecting SS closer to the SS of the pseudo-observed data), in agreement with previous studies [51], and evaluated the distance between the estimated and true parameter values. Next, we estimated the parameters from the observed data using the methodology previously described for the pseudo-observed data.

## 3. Results

In this section, we present the results of the phylogenetic analysis of SEA populations followed by the selection of the best-fitting evolutionary scenario and the population genetics parameters estimation.

### 3.1. Phylogenetic Inference Suggests Genetic Admixture between SEA Populations

**Figure 2:**
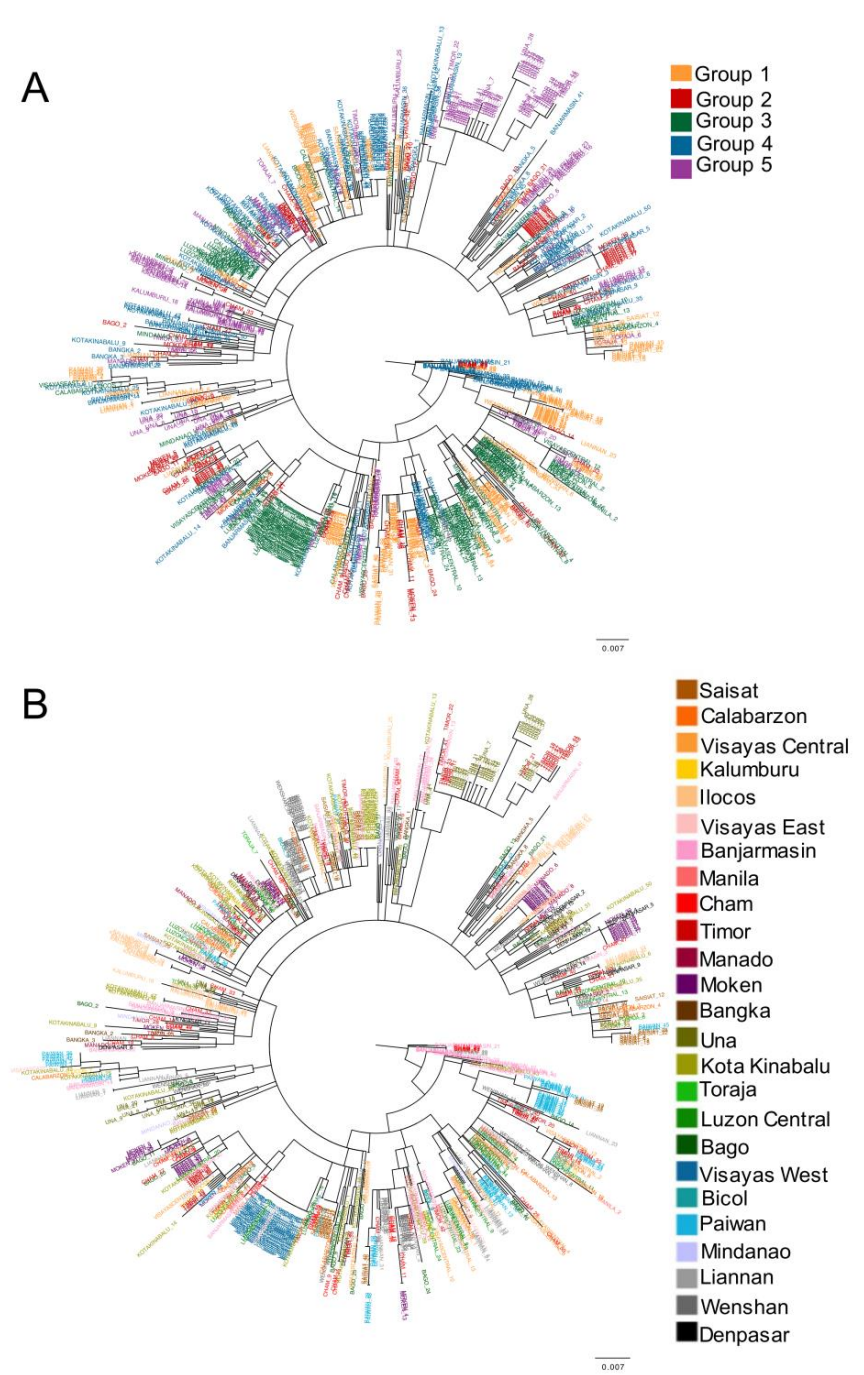
Phylogenetic tree reconstructed from the genetic data. Phylogenetic tree reconstructed with maximum likelihood, with sample names colored according to their corresponding geographic group (**A**) and studied population (**B**) (Figure 1 and Table S1).

The reconstructed phylogenetic tree revealed remarkable genetic admixture among SEA groups (Figure 2A) and populations (Figure 2B), regardless of the geographic distance among groups and populations. This genetic admixture could be caused by both LDD and the LGP (both can favor migration among SEA populations), which is explored in the following subsections.

### 3.2. The Sea Level Variation Caused by the LGP and LDD Fits with the Observed Genetic Diversity in SEA Populations

The goodness-of-fit analysis indicated that the spatially explicit computer simulations, especially those considering the sea level variation caused by the LGP and LDD, can mimic the observed data (Figure S6, Supplementary Material). Indeed, the applied ABC methods (*mnlogistic* and *neuralnet*, see Section 2) identified every studied evolutionary scenario with acceptable error (Table S4 and Figure S7, Supplementary Material). Next, the evolutionary scenario that considers both the sea level variation and LDD (scenario *LGP&LDD*) best fitted the observed data, showing posterior probabilities of 0.87 and 0.70 under the *mnlogistic* and *neuralnet* methods, respectively (Table 1A). Indeed, pairwise comparisons between the evolutionary scenarios and the observed data also revealed that the evolutionary scenarios accounting for the LGP and LDD best fit the observation (Table 1B–D).

### 3.3. Evolutionary Parameters Estimation Suggests Rapid Migration Favored by Temporarily Exposed Lands Due to the LGP and LDD

The multiple linear regression method for parameters estimation showed that, for all the parameters under study, the true value always fell well within the 50% highest posterior density interval (HPDI) of the estimates (including mode, median and mean of the posterior distributions) from the 100 pseudo-observed data points (see Section 2 and Table S5; Supplementary Material), which has been considered as an acceptable estimation error [17,51].

Next, the parameter estimates for the observed data are presented in Table 2 and Figure S8 (Supplementary Material). The estimated time of the onset of the range expansion was around 64,000 ya (95% HPDI: 60,325–69,475) and agrees with previous estimates [17], as well as with current genetic [33] and archaeological evidence [10]. Regarding the population size at the onset of the expansion, interestingly, we found a large population size (around 40,000 with 95% HPDI: 25,025–70,606), which agrees with evidence of large expanding populations in the region [41]. The estimated population growth rate (around 0.6 with 95% HPDI: 0.40–0.95) agrees also with estimates from previous studies of hunter–gatherer populations from Eurasia [16] and the Philippines [17]. We separately estimated the migration rate in permanent and temporary (regions exposed due to the lowering sea level caused by the LGP) lands. Interestingly, we did not find significant differences among them (Table 2) and both estimates agree with previous studies [16,17]. This finding suggests that temporarily exposed land regions were used for migration to a similar extent than permanent land regions and, therefore, the LGP could have favored additional migration through those temporarily available lands. A similar result was obtained for the carrying capacity, which was also separately estimated for permanent and temporary lands. In particular, the 95% HPDI of the posterior distributions for the carrying capacity in permanent (1001–3727) and temporary (1049–3794) lands overlap; however, the mean, median and mode of the posterior distributions were higher for temporary lands (Table 2). This suggests that temporarily exposed lands derived from the lowering sea level caused by the LGP provided resources that could increase population sizes before the re-flooding of such regions. Finally, the inferred mutation rate (around 4 × 10^−6^, with 95% HPDI: 1.051 × 10^−7^–9.213 × 10^−6^) agrees with estimates from previous studies [17,52] and the same occurred with the estimated proportion of LDD events (around 0.027, with 95% HPDI: 0.01–0.024), which are in agreement with previous studies [16,17].

## 4. Discussion

The influence of the LGP on the first expansions of EMHs has been a subject of debate by scientists, particularly because this environmental process changed the available land and the vegetation distribution [14]. In this light, most of studies focused on the demographic and genetic consequences of range contractions [1,2,3] and habitat fragmentation induced by the extended ice sheets and deserts caused by the LGP [6,7]. By contrast, here we explored whether the temporarily available lands derived from the lowering sea level caused by the LGP, together with LDD (although the presence of LDD could be expected according to previous studies [4,46]), influenced the expansion of EMHs throughout SEA, and if they are required to understand the currently observed genetic patterns in this region.

We found that the colonization of SEA was affected by the sea level variation caused by the LGP and by migration events with LDD (Table 1), which favored a rapid range expansion (more rapid than the other evaluated evolutionary scenarios (Figures S1–S4) and in agreement with previous studies [23]), as well as a large population admixture and genetic diversity throughout SEA (Figure 2). These genetic consequences are hardly surprising since it is known that LDD increases genetic diversity and prevents differentiation between populations [16,46]. It also is known that the low sea level caused by the LGP produced temporarily available lands that connected or made it possible to reach previously isolated landmasses [14]. In this concern, Li and Li [53] found that these sea level changes in SEA produced gene flow among populations of diverse species that resulted in an increase of population genetic diversity. Another example involves populations of an Indonesian bat species, where the genetic distance between populations living in islands unconnected during the LGP (i.e., Timor and Sumba) is larger than the genetic distance between populations living in islands connected during that period (i.e., Timor and Alor) [54]. Concerning EMHs, our results suggest that the sea level variation could have favored the expansion through the region and contributed to the large population admixture that is currently observed in the local populations.

The estimated demographic and genetic parameters generally agreed with estimates from previous studies [10,12,14,23,41] and point to a rapid expansion throughout SEA by large Paleolithic populations of our species around 65,000 ya. This rapid expansion could be explained by the presence of LDD events (we found that around 3% of migration events could be LDD and it is known that EMHs expanded from SEA additionally using maritime technologies [14]) and by the low sea level caused by the LGP. Of course, other human species could have coexisted with EMHs in SEA during that period [10] but in the present study our models had to assume a lack of those possible interactions.

Next, we discuss the population genetics consequences derived from using temporarily available lands (lands exposed during the LGP), in comparison with permanent lands. We found that the migration rate estimated from temporarily available lands was similar to the migration rate estimated from permanent lands (Table 2). This finding suggests that temporary lands were used for migration as much as permanent lands and, therefore, supports that the LGP favored gene flow between SEA populations. Moreover, we also found that the carrying capacity estimated from temporarily available lands could be even higher (although HPDI overlaps, see Table 2) than the carrying capacity estimated from permanent lands. This suggests that lands exposed by the LGM could have provided additional resources to the expanding populations and contributed to the population increase. The vegetation in SEA during the LGP included the presence of tropical rainforests and “savanna” covering the Sunda shelf and connecting multiple SEA islands, which could favor the expansion of EMHs [13]. In addition, we believe that shellfishing and other beachcombing activities [23] could be relevant in temporary lands. It is noticeable that SEA was occupied by EMH populations with advanced knowledge about plants, animals, beachcombing and orientation skills [10]. Actually, our estimated carrying capacity (focused on SEA) was higher than the carrying capacity estimated for Eurasian populations [16], suggesting that SEA presented considerable resources to promote a rapid population increase. Altogether, our findings show that temporarily available lands exposed during the LGP were relevant for the expansion and admixture of SEA populations.

Analyzing SEA as an illustrative example, we show here that past environmental fluctuations affected the first expansion of EMHs and could produce genetic consequences that can be detected (to a certain extent) today. Despite in multiple regions of the world the LGP produced a decline in genetic diversity due to range contractions and habitat fragmentation (caused by the increase of ice sheets and deserts), we found the opposite situation in SEA, where the LGP (through the lowering sea level) and LDD favored the expansion and admixture of modern human populations. Consequently, we believe that studies on the evolution of modern humans should consider, as much as possible, past environmental changes.

## 5. Conclusions

It is known that the range contractions, range shifts and habitat fragmentation derived from the large ice sheets during the last glacial period overall produced a genetic diversity decline in Paleolithic populations. However, the last glacial period, together with long-distance dispersal, could increase the genetic diversity in some regions of the world, such as Southeast Asia. In particular, the temporarily available lands caused by the lowering sea level allowed active human migration and produced large carrying capacities, likely from shellfishing and beachcombing activities. In general, we conclude that environmental factors should be considered to properly understand and model the evolution of our species.

## Figures and Tables

**Table 1:** Fitting of the studied evolutionary scenarios with the observed genetic data. The table shows the fitting (posterior probability estimated with the *mnlogistic* and *neuralnet* methods) of each evolutionary scenario with the observed data in diverse evaluations: (A) all the studied evolutionary scenarios together; (B) the evolutionary scenarios *LDD* and *LGP*; (C) the evolutionary scenarios *LGP* and *LGP****&****LDD*; (D) the evolutionary scenarios *LDD* and *LGP****&****LDD*. Additional evaluations with the evolutionary scenario *NONE* are not included because this scenario always produced the worst fitting. The best-fitting evolutionary scenario (and its posterior probability) for each evaluation is presented in bold.

Evaluated Evolutionary Scenarios	Posterior Probability							
	*Mnlogistic*				*Neuralnet*			
(A) *NONE* vs. *LGP* vs. *LDD* vs. *LGP&LDD*	0.000	0.000	0.130	**0.869**	0.002	0.003	0.130	**0.701**
(B) *LDD* vs. *LGP*	**0.989**		0.011		**1.000**		0.000	
(C) *LGP* vs. *LGP&LDD*	0.001		**0.999**		0.001		**0.999**	
(D) *LDD* vs. *LGP&LDD*	0.091		**0.909**		0.171		**0.829**	

**Table 2:** Population genetic parameters estimated under the best-fitting evolutionary scenario (*LGP&LDD*). Note that the migration rate and carrying capacity were separately estimated for demes belonging to permanent (*m* and *K*) and temporary (*m_temp* and *K_temp*) lands. For each parameter the table presents the mode, mean, median and **95% HPDI** of the posterior distribution. A graphical representation of these posterior distributions is provided in Figure S8.

Parameter	Mode	Mean	Median	95% HPDI
**Time of onset of the expansion** ***(T)***	64,650 ^†^	64,900 ^†^	64,875 ^†^	60,325–69,475 ^†^
**Population size at the onset of the expansion (*N*)**	37,462	48,570	48,173	25,025–70,606
**Population growth rate (*r*)**	0.5223	0.671	0.659	0.400–0.946
**Migration rate (*m*)**	0.222	0.246	0.243	0.200–0.291
**Migration rate in temporary lands (*m_temp*)**	0.219	0.247	0.246	0.200–0.292
**Carrying capacity (*K*)**	1849	2387	2336	1001–3727
**Carrying capacity in temporary lands (*K_temp)***	2660	2466	2465	1049–3794
**Mutation rate (*μ*)**	3.904 × 10^−6^	4.759 × 10^−6^	4.665 × 10^−6^	1.051 × 10^−7^–9.213 × 10^−6^
**LDD proportion (*LDDprop*)**	0.027	0.029	0.029	0.011–0.047

^†^ Time is shown in years.

## Data Availability

The studied real data (details in Table S1), input and output files are publicly available from the Zenodo repository http://doi.org/10.5281/zenodo.5515856 (accessed on 12 February 2022) [55].

## Supplementary Materials

The following supporting information can be downloaded at: https://www.mdpi.com/article/10.3390/genes13020384/s1, Information on the spatially explicit computer simulations; Table S1: Samples location, sample size and references of the studied data; Table S2: Prior distributions for the demographic and genetic parameters applied in the computer simulations; Table S3: Summary statistics and their estimation for the observed dataset; Table S4: Power of the applied ABC methods to select among the studied evolutionary scenarios; Table S5: Power of the ABC method in parameters estimation; Figures S1–S4: Illustrative examples of the simulated evolutionary scenarios; Figure S5: Boxplots displaying the distribution of the selected summary statistics computed from the data simulated under each evolutionary scenario; Figure S6: Goodness-of-fit of the data simulated under every evolutionary scenario; Figure S7: Power of the ABC multinomial logistic regression and neural networks based methods for selecting an evolutionary scenario among the studied evolutionary scenarios; Figure S8: Prior and posterior distributions of the parameters estimated under the best-fitting evolutionary scenario (LGP&LDD) [16,17,18,19,21,23,24,25,26,27,28,29,30,31,32,33,40,41,42,46,47,51,52,56,57,58,59,60,61,62,63].
