# Supplementary material for: Consequences of the Last Glacial Period on the Genetic Diversity of Southeast Asians
Source: Genes (Basel). 2022 Feb 21;13(2):384. doi: 10.3390/genes13020384 (PMC8871837; doi:10.3390/genes13020384)
Supplement: Supplementary file 1 [file genes-13-00384-s001.zip › genes-1585267-supplementary.pdf]

Supplementary Material

Consequences of the Last Glacial Period on the Genetic Diversity of Southeast Asians

Spatially explicit computer simulations

We performed the spatially explicit computer simulations with the framework *SPLATCHE3* [1]. This framework simulates multiple alignments of DNA sequences under evolutionary scenarios that account for environmental heterogeneity over time and space. Note that spatially explicit models of evolution are more realistic than models that ignore the landscape and environment [2,3]. *SPLATCHE3* comprises two main processes: (*a*) A forward-in-time phase that includes the simulation of the demographic history of the population expanding over a landscape (which can be obtained with geographic information systems [4]) under a 2D stepping-stone migration model [5] (but it can also simulate long-distance dispersal, see later) and according to user-specified demographic parameters. (*b*) A backward-in-time phase that considers the demographic history previously simulated to obtain a coalescent tree for a user-specified sample (including its size and location). Then it performs the simulation of genetic data (i.e., DNA sequences) along the simulated coalescent tree considering a mutation rate μ.

We applied a demographic model implemented in *SPLATCHE3* [1], in which the population growth follows a standard logistic curve, controlled by the carrying capacity and growth rate [1]. The number of emigrants in the four directions was assumed constant (isotropic migration) to avoid overparameterization [1]. The demographic model allows the user to define a proportion of migrants (*LDDprop*) moving through long-distance dispersal (LDD) within a user-specified maximum migration distance (we applied 500 km following Arenas et al. [6]) under the LDD model implemented by Ray and Excoffier [7]. Importantly, the evolutionary model considered that demes without resources (carrying capacity = 0) cannot allocate individuals. Under that model, we designed a total of 4 evolutionary scenarios based on considering and/or ignoring the sea level variation induced by the last glacial period (LGP) and migration through LDD. We adapted the 2D map of Southeast Asia (SEA) of 44,064 demes (216×204, each deme corresponds to 25×25 km) provided by Arenas et al. [6]. A total of 11,379 demes were permanent land (common to all evolutionary scenarios) and a total of 6,763 demes were temporarily exposed land in scenarios that consider the LGP (scenarios

LGP and *LGP&LDD*, for the remaining scenarios they were permanent water regions). The applied demographic and genetic parameters were drawn from uniform distributions based on previous studies (see Table S4 for details). Briefly, a population (comprising *N* individuals) started to expand from current Bangladesh at time *T* years ago under a population growth rate *r*. For scenarios that ignore the LGP (scenarios *NONE* and *LDD*), the migration rate (*m*) and carrying capacity (*K*) were constant over time and space. For scenarios that consider the LGP (scenarios *LGP* and *LGP&LDD)*, the migration rate and carrying capacity varied through time and space (different rates for permanent and temporarily exposed lands). In particular, temporary land demes included a migration rate and carrying capacity (*m_temp* and *K_temp*, respectively) different to the migration rate and carrying capacity of permanent land demes. When temporary land demes were submersed, their migration rate and carrying capacity were set to 0. In order to model short maritime migrations (without LDD), that have been proposed in [6], we implemented narrow land corridors (Figures S1-S4) with low carrying capacity (*K*=25) and migration rate (*m*=0.0125 towards every direction) [6].

Table S1. Sample location, sample size and references of the studied data. The samples belong to individuals of diverse SEA populations and were arranged in 5 groups (first column) according to their geographic distribution (see the main text). The column headed *Sample size* refers the number of individuals per location. The column headed *References* cites the studies from which the genetic sequences were retrieved.

Group	Country (island)	Region/Population	Sample size	References
Group 1	China	Liannan	35	[8]
		Wenshan	39	[8]
	Taiwan	Saisiat	40	[9]
		Paiwan	50	[10]
Group 2	Myanmar	Bago	28	[11]
	Thailand	Moken	40	[12]
	Vietnam	Cham	50	[13]
Group 3	Philippines	Ilocos	13	[6,14–16]
		Central Luzon	24	[6]
		Manila	17	[6]
		Calabarzon	20	[6]
		Bicol	10	[6]
		Western Visayas	30	[6]
		Eastern Visayas	16	[6]
		Central Visayas	14	[6]
		Mindanao	14	[6]
Group 4	Malaysia (Borneo)	Kota Kinabalu	50	[17]
	Indonesia (Sumatra)	Bangka	10	[18]
	Indonesia (Borneo)	Banjarmasin	50	[14,16,19]

	Indonesia (Bali)	Denpasar	19	[17]
Group 5	Indonesia (Sulawesi)	Manado	10	[17]
		Toraja	10	[17]
	Indonesia (Papua)	Una	50	[20]
	Timor-Leste	Timor	50	[21]
	Australia	Kalumburu	32	[22]

Table S2. Prior distributions for the demographic and genetic parameters applied in the computer simulations. For every parameter (its abbreviation is shown in parenthesis), the table includes the applied prior distribution (note that we specified uniform prior distributions to all the parameters following previous works [23,24]), the studied evolutionary scenarios that consider the parameter (*All* indicates that the parameter is used in all the studied evolutionary scenarios) and the references that support the applied parameter values of the prior distribution.

Parameter	Prior distribution	Studied scenario	Reference
**Time of the beginning of the expansion (*T*)**	Uniform (60,000 – 70,000)†	All	[6,18,25]
**Initial population size (*N*)**	Uniform (25,000 – 75,000)	All	[6,26,27]
**Population growth rate (*r*)**	Uniform (0.4 – 1.0)	All	[6,28,29]
**Migration rate (*m*)**	Uniform (0.2 – 0.3)	All	[6,28]
**Migration rate of temporary land (*m_temp*)**	Uniform (0.2 – 0.3)	*LGP* and *LGP&LDD*	[6,28]
**Carrying capacity (*K*)**	Uniform (1,000 – 4,000)	All	[6]
**Carrying capacity of temporary land (*K_temp*)**	Uniform (1,000 – 4,000)	*LGP* and *LGP&LDD*	[6]
**Mutation rate (μ)**	Uniform (1×10^{-15} – 1×10^{-5})	All	[6,30–32]
**LDD proportion (*LDDprop*)**	Uniform (0.01 – 0.05)	*LDD* and *LGP&LDD*	[28]

†Time is shown in years.

Table S3. Summary statistics and their estimation for the observed dataset. The applied summary statistics (details about its selection in the main text) include genetic differentiation (F_{ST}) between populations located in the most northwestern and southeastern regions (entries 1-9), decay of the genetic differentiation (F_{ST}) between the Bago population (the most northwestern population) and all the other populations with the geographic distance between them (entry 10) and, decay of the nucleotide diversity of all populations with the distance to the geographic origin of the expansion (entry 11).

Entry	Summary Statistics	Value in observed data
**1**	Genetic differentiation between populations Bago and Timor-Leste (*FST_Bago_Timor*)	0.109
**2**	Genetic differentiation between populations Bago and Una (*FST_Bago_Una*)	0.003
**3**	Genetic differentiation between populations Bago and Kalumburu (*FST_Bago_Kalumburu*)	0.117
**4**	Genetic differentiation between populations Wehnshan and Timor-Leste (*FST_Wehnshan_Timor*)	0.007
**5**	Genetic differentiation between populations Wehnshan and Una (*FST_Wehnshan_Una*)	0.021
**6**	Genetic differentiation between populations Wehnshan and Kalumburu (*FST_Wehnshan_Kalumburu*)	0.034
**7**	Genetic differentiation between populations Liannan and Timor-Leste (*FST_Liannan_Timor*)	0.004
**8**	Genetic differentiation between populations Liannan and Una (*FST_Liannan_Una*)	-0.009
**9**	Genetic differentiation between populations Liannan and Kalumburu (*FST_Liannan_Kalumburu*)	0.043

**10**	Decay of the genetic differentiation between the Bago population and all the other populations with their geographic distance (*slope_FSTtoBago*)	1.537×10^{-7}
**11**	Decay of the nucleotide diversity per population with the geographic distance to the location of the origin of the expansion (*slope_allPi*)	-1.940×10^{-4}

Table S4. Power of the applied ABC methods to select among the studied evolutionary scenarios. The table shows the proportion (between 0 and 100) of pseudo-observed simulations in a cross-validation analysis to predict an evolutionary scenario with the two applied ABC methods (multinomial logistic regression (*Reg*) and neural networks based (*Nn*)). Each row consists in 100 cross-validation of pseudo-observed simulations belonging to every evolutionary scenario. Each column consists in the predicted evolutionary scenario for the pseudo-observed simulations. Identification of the correct scenario is shown in bold (main diagonal). A graphical representation of this table is provided in Figure S7.

Predicted / Simulated	***NONE***		***LGP***		***LDD***		***LGP&LDD***	
	Reg	*Nn*	*Reg*	*Nn*	*Reg*	*Nn*	*Reg*	*Nn*
NONE	**100**	**100**	0	0	0	0	0	0
LGP	7	2	**88**	**96**	0	2	3	0
LDD	0	0	0	2	**72**	**76**	28	22
LGP&LDD	3	0	0	1	28	23	**72**	**76**

Table S5. Power of the ABC method in parameters estimation. For every parameter inferred from the pseudo-observed data we computed the distance between the true and the estimated value (including median, mean and mode). The dashed line indicates that the estimated parameter value is equal to the true parameter value (estimation error = 0). This evaluation is only applied to the previously selected evolutionary scenario (*LGP&LDD*).

**Parameter**	**Estimation error**		
	**Mode**	**Mean**	**Median**
**Time of the beginning of the expansion (*T*)**	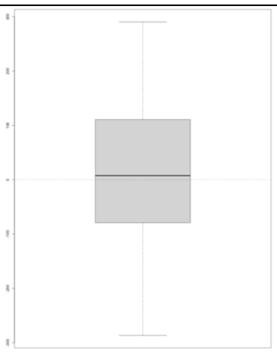	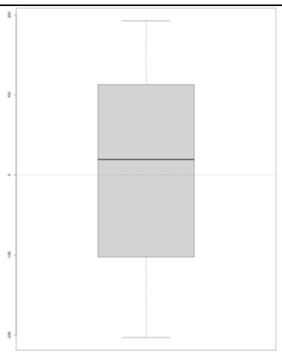	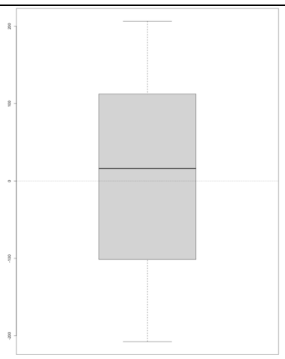
**Initial population size (*N*)**	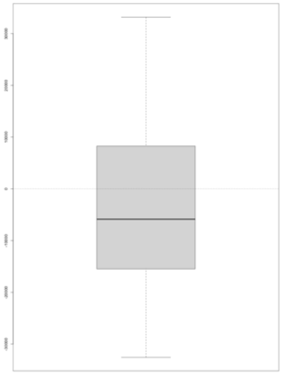	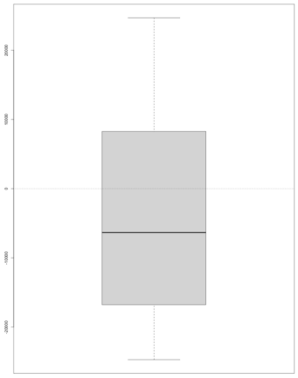	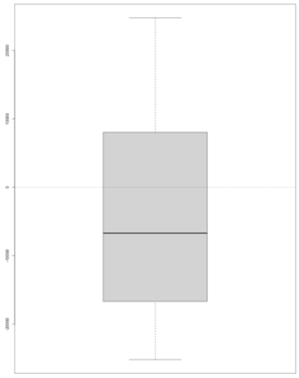
**Population growth rate (*r*)**	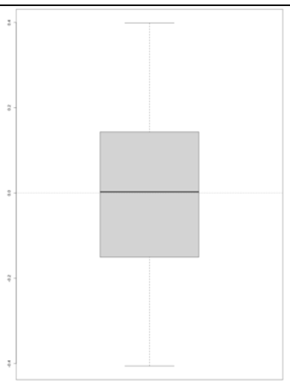	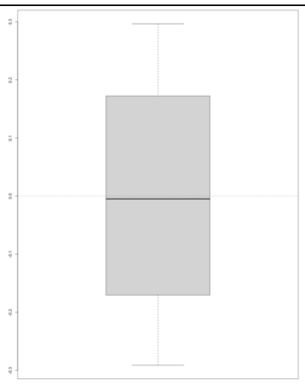	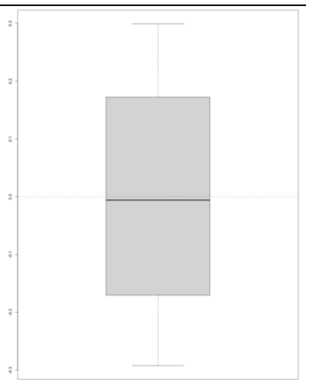

**Migration rate (*m*)**	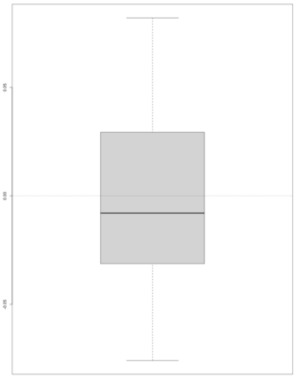	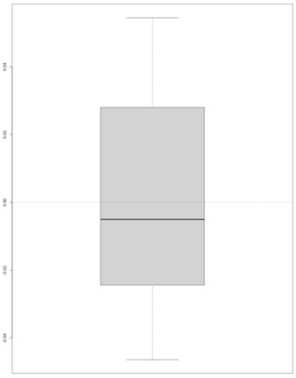	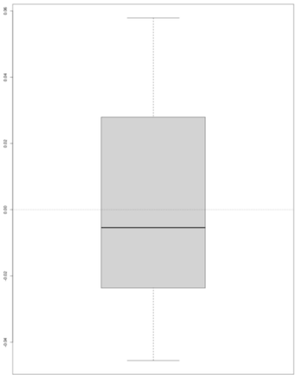
**Migration rate of temporary land (*m_temp*)**	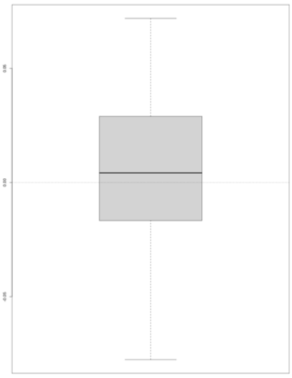	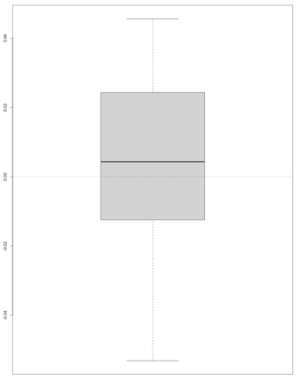	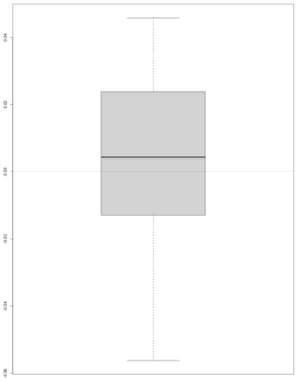
**Carrying capacity (*K*)**	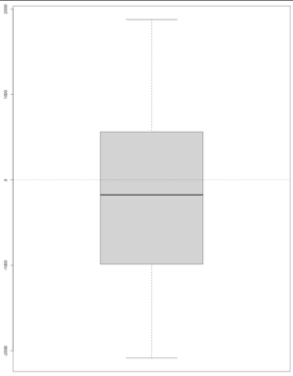	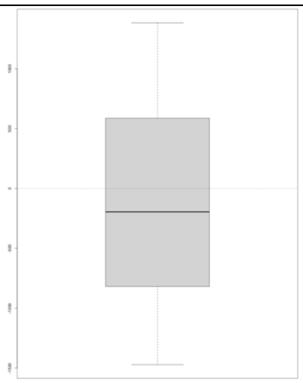	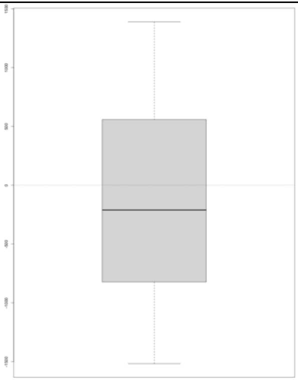

**Carrying capacity of temporary land (*K_temp*)**	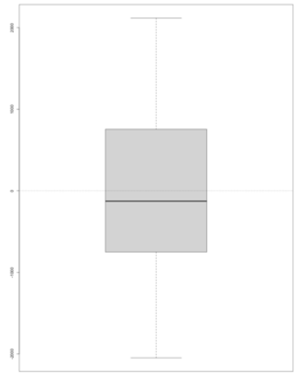	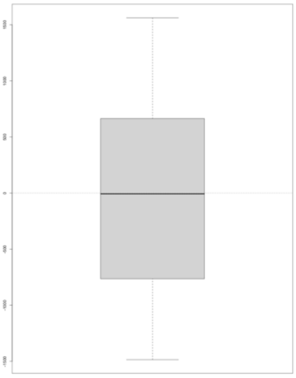	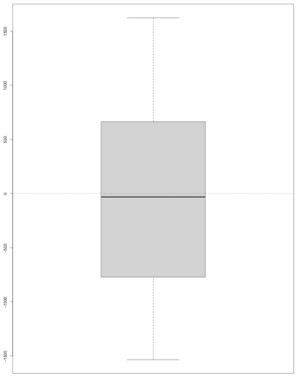
**Mutation rate (*μ)***	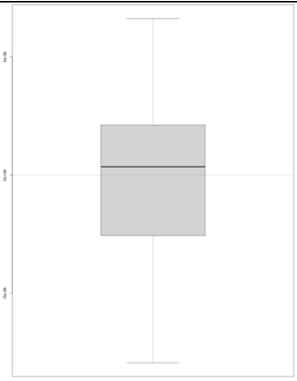	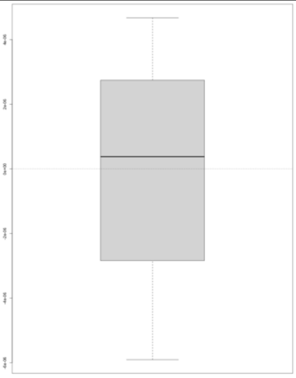	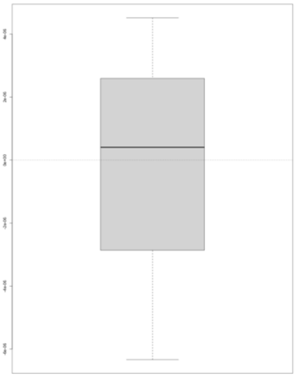
**LDD proportion (*LDDprop)***	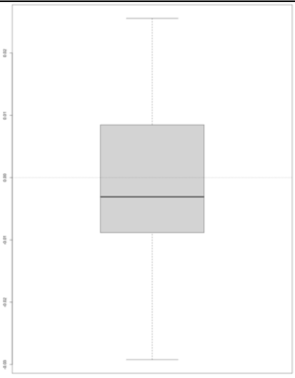	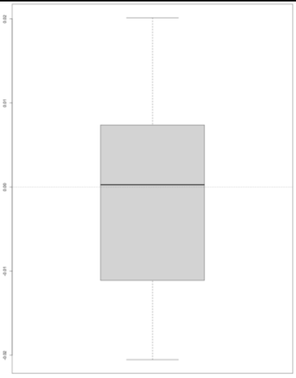	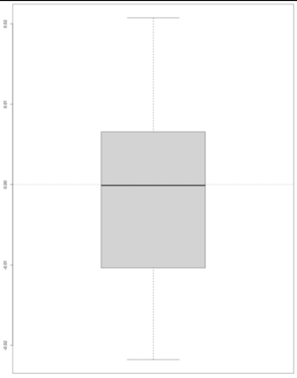

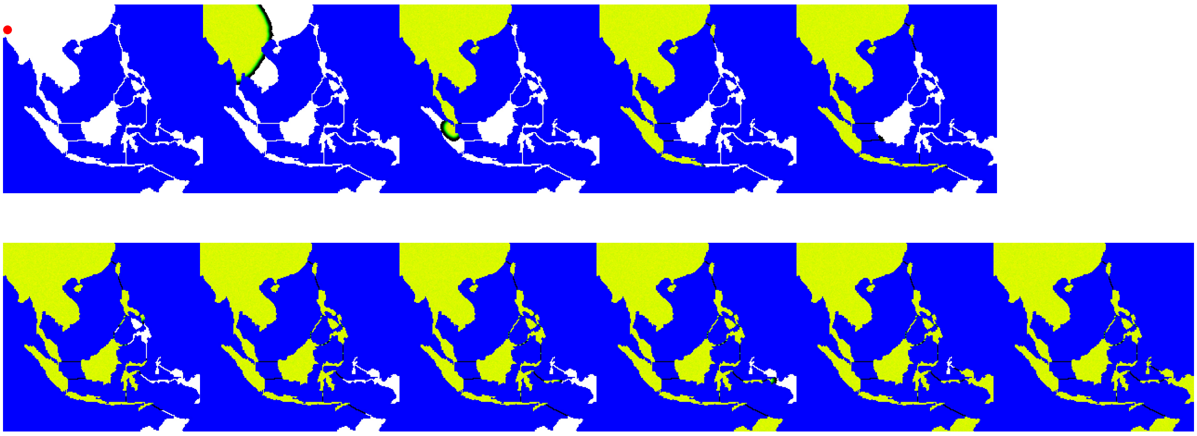

Figure S1. Illustrative example of the range expansion over SEA under the evolutionary scenario that ignores sea level variation and considers gradual migration (scenario *NONE*). The colonization starts from a single deme located at the northwest of SEA (red point in the first snapshot). Under this evolutionary scenario the sea level is constant and equal to the current level. Green and white regions are colonized and not colonized regions, respectively. Screenshots correspond to every 200 generations (from the left to the right).

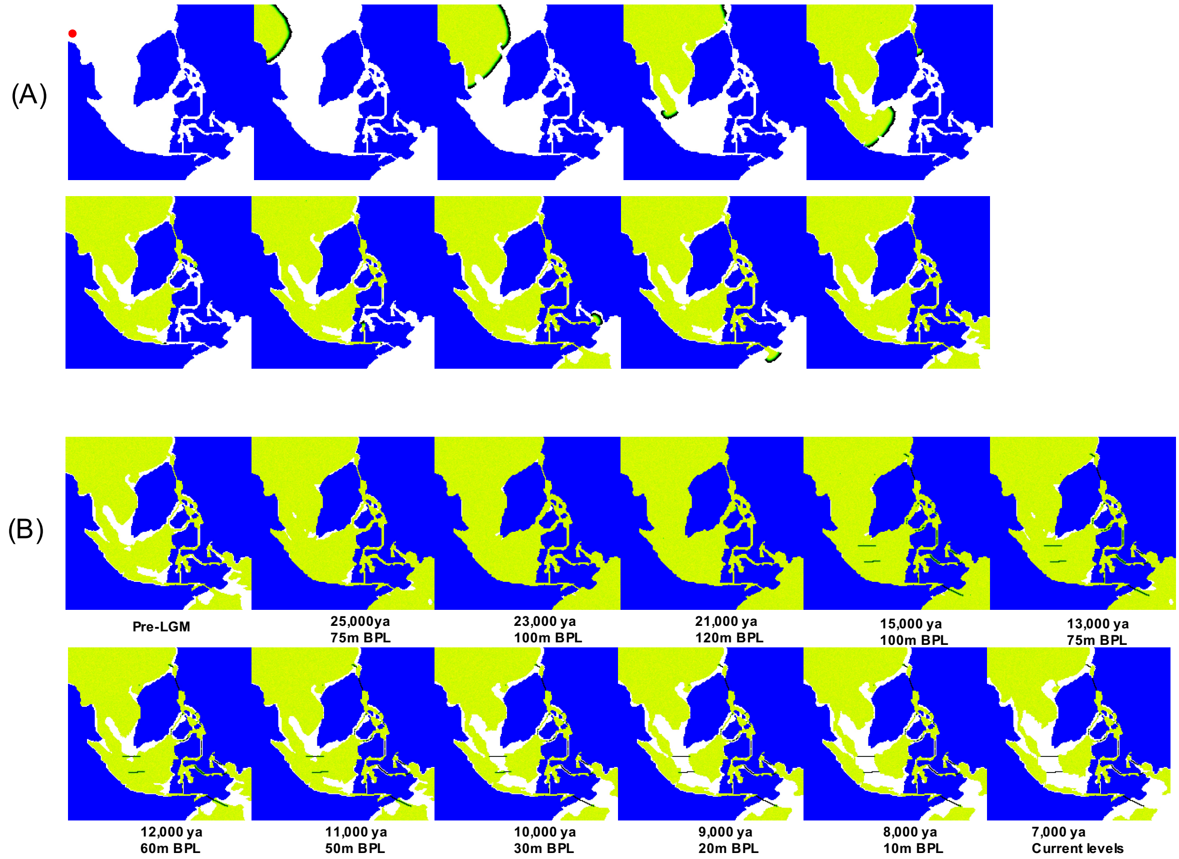

Figure S2. Illustrative example of the range expansion over SEA under the evolutionary scenario that considers sea level variation and considers gradual migration (scenario *LGP*). (A) The colonization starts from a single deme located at the northwest of SEA (red point in the first snapshot) during the LGP, with sea level approximately 50 m BPL. Green and white regions are colonized and not colonized regions, respectively. Screenshots correspond to every 100 generations (from left to right). (B) Sea level variation after the colonization of SEA. Green regions and white regions are colonized and not colonized (submersed land) regions, respectively. The timing and sea level for each snapshot (from the left to the right) are included in the figure.

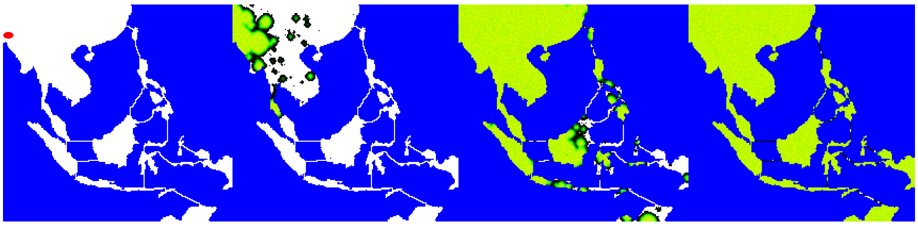

Figure S3. Illustrative example of the range expansion over SEA under the evolutionary scenario that ignores sea level variation and considers gradual migration together with LDD (scenario *LDD*). The colonization starts from a single deme located at the northwest of SEA (red point in the first snapshot). Under this evolutionary scenario the sea level is constant and equal to the current level. Green and white regions are colonized and not colonized regions, respectively. Screenshots correspond to every 50 generations (from the left to the right).

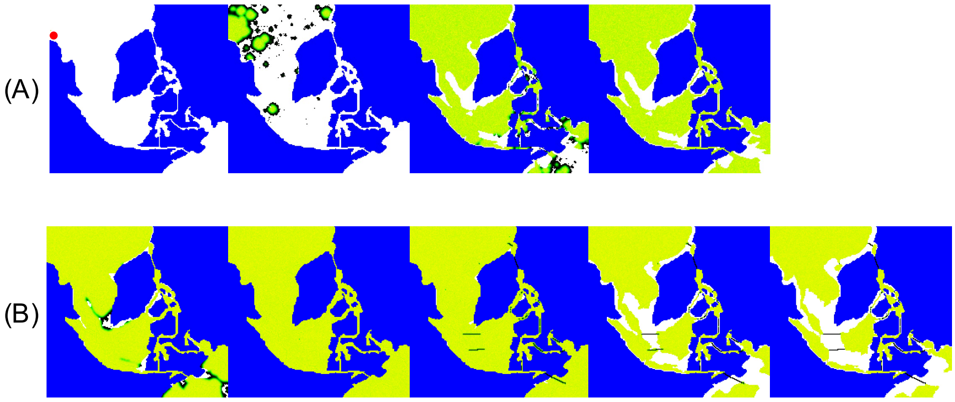

Figure S4. Illustrative example of the range expansion over SEA under the evolutionary scenario that considers sea level variation and considers gradual migration together with LDD (scenario *LGP&LDD*). (A) The colonization starts from a single deme located at the northwest of SEA (red point in the first snapshot) during the LGP, with sea level approximately 50 m BPL. Green regions are colonized regions and white regions are areas without populations. Screenshots correspond to every 50 generations (from the left to the right). (B) Sea level variation after the colonization of SEA. Green regions and white regions are colonized and not colonized (submersed land) regions, respectively. Screenshots correspond to every 50 generations after the beginning of the LGM (from the left to the right). The detailed timing and sea level variation after the colonization of SEA is detailed in Figure S2B.

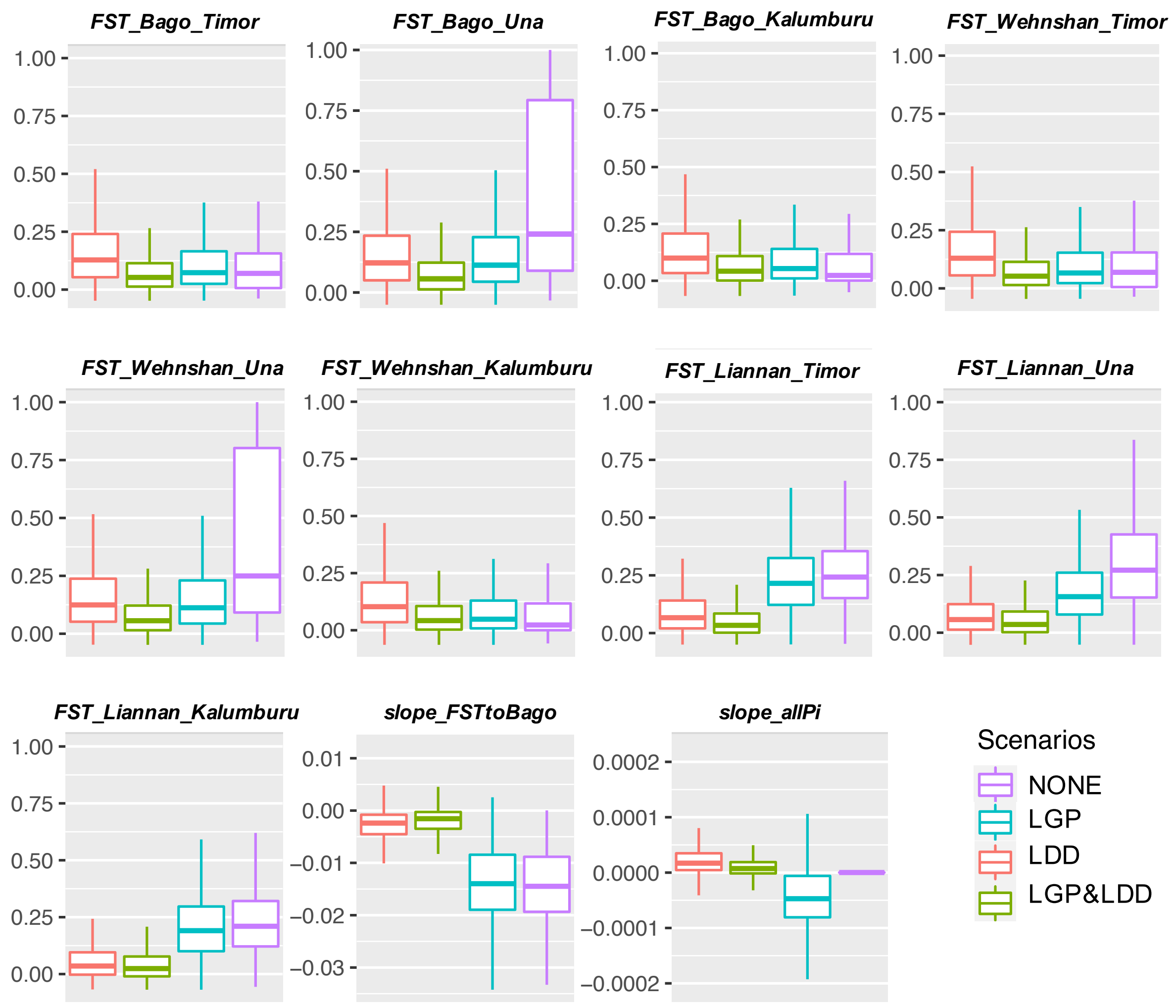

Figure S5. Boxplots displaying the distribution of the selected summary statistics computed from the data simulated under each evolutionary scenario. These SS were selected considering that they can distinguish, at least partially, between the studied evolutionary scenarios. The meaning of each SS is explained in the Table S3.

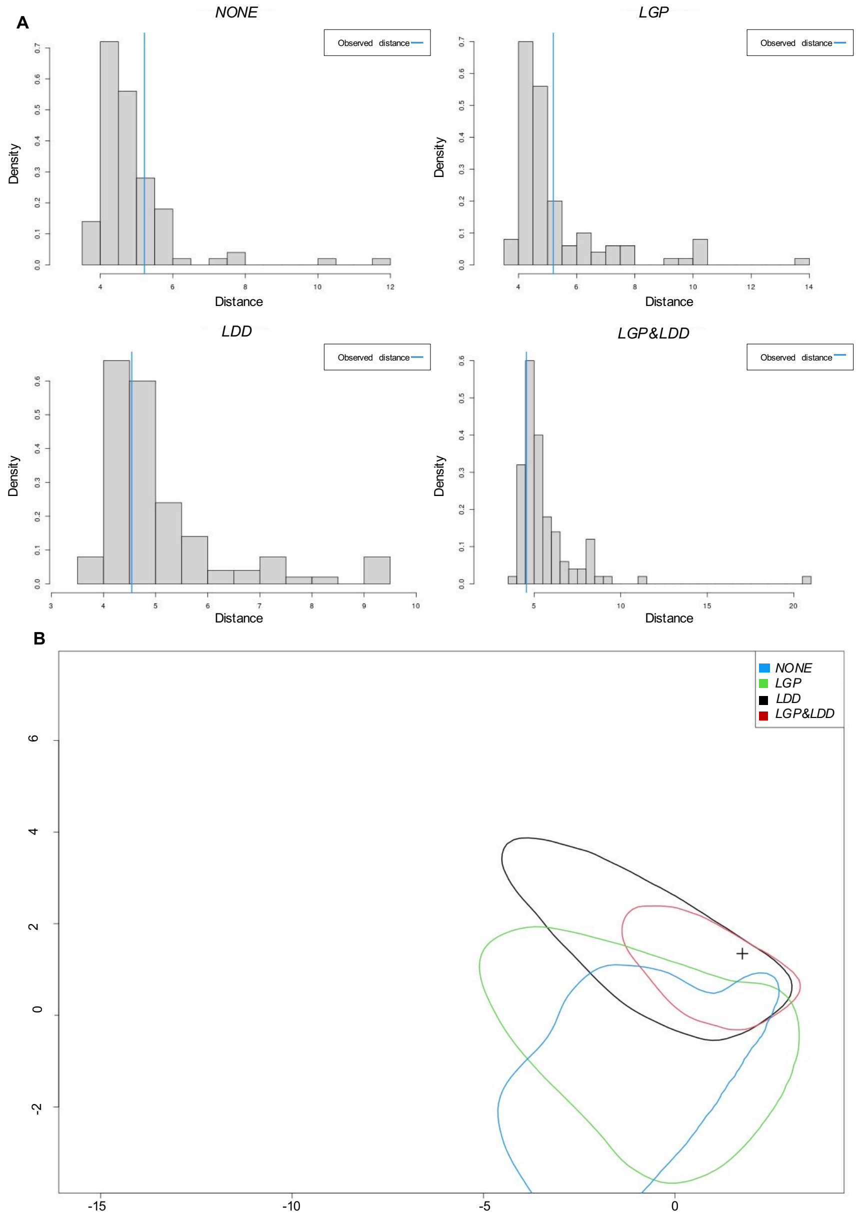

Figure S6. Goodness-of-fit of the data simulated under every evolutionary scenario. (A) Evaluation of the fitting of the simulated data with the observed data by the distance between the distribution of the SS of the simulated data (histogram) and the SS from the observed data (blue vertical line). These analyses agree with the posterior probability of each evolutionary scenario (Table 1). (B) Evaluation of the fitting of the simulated data based on the first two components obtained with a principal component analysis. The plot displays the 90% of datasets simulated under each scenario. Note that the projection of the observed SS (black cross) falls within the scenarios *LDD* and *LGP&LDD*, suggesting a better fitting with these scenarios.

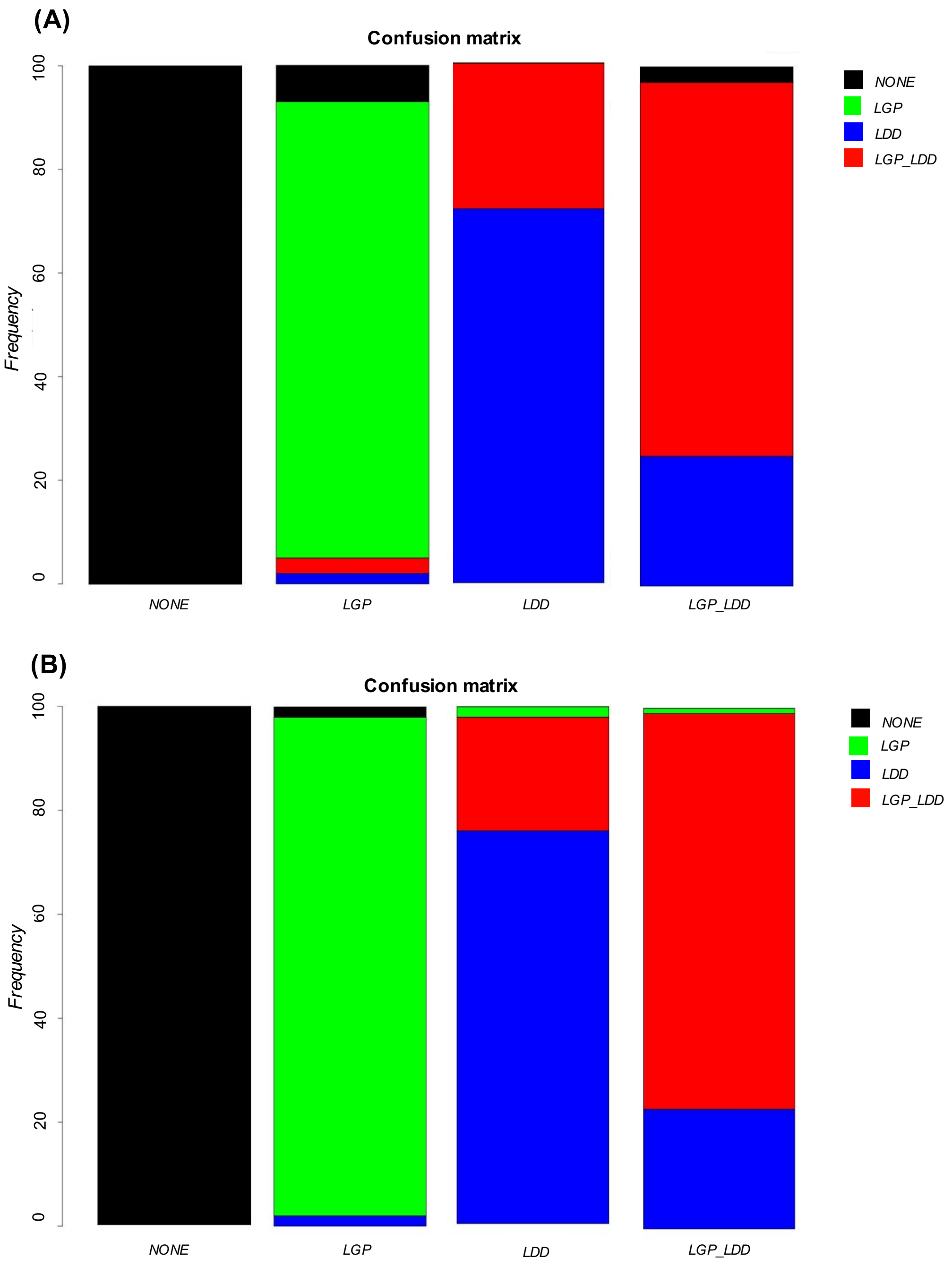

Figure S7. Power of the ABC multinomial logistic regression and neural networks based methods for selecting an evolutionary scenario among the studied evolutionary scenarios. Each bar corresponds to the proportion (between 0 and 100) of pseudo-observed simulations in a cross-validation analysis to predict an evolutionary scenario with the two applied ABC methods, multinomial logistic regression (A) and neural networks based (B). The color indicates the scenario predicted for each simulated dataset (see legend). Note that if the evolutionary scenario is correctly selected for every simulated dataset, then each bar will only have one color (corresponding to the true evolutionary scenario).

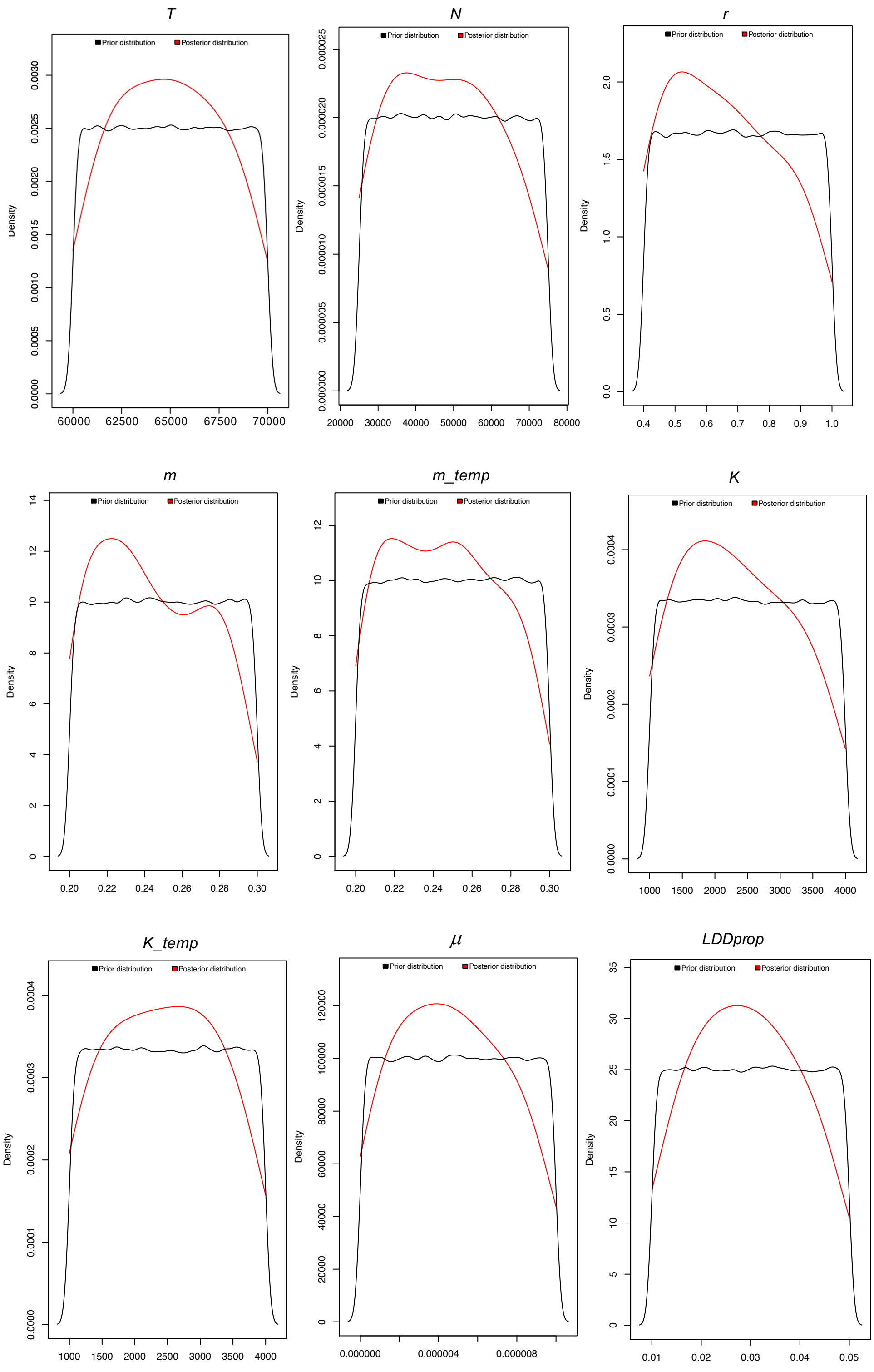

Figure S8. Prior and posterior distributions of the parameters estimated under the best fitting evolutionary scenario (*LGP&LDD*). The prior and posterior distributions are shown in black and red, respectively. Additional information about these posterior distributions is shown in Table 2.

References

1. Currat, M.; Arenas, M.; Quilodran, C.; Excoffier, L.; Ray, N. SPLATCHE3: Simulation of Serial Genetic Data under Spatially Explicit Evolutionary Scenarios Including Long-Distance Dispersal. *Bioinformatics* **2019**, *35*, 4480–4483.
2. Epperson, B.K.; Mcrae, B.H.; Scribner, K.; Cushman, S.A.; Rosenberg, M.S.; Fortin, M.-J.; James, P.M.A.; Murphy, M.; Manel, S.; Legendre, P.; et al. Utility of Computer Simulations in Landscape Genetics. *Mol. Ecol.* **2010**, *19*, 3549–3564.
3. Benguigui, M.; Arenas, M. Spatial and Temporal Simulation of Human Evolution. Methods, Frameworks and Applications. *Curr. Genomics* **2014**, *15*, 245–255.
4. Leempoel, K.; Duruz, S.; Rochat, E.; Widmer, I.; Orozco-terWengel, P.; Joost, S. Simple Rules for an Efficient Use of Geographic Information Systems in Molecular Ecology. *Front. Ecol. Evol.* **2017**, *5*, 33.
5. Kimura, M.; Weiss, G.H. The Stepping Stone Model of Population Structure and the Decrease of Genetic Correlation with Distance. *Genetics* **1964**, *49*, 561–576.
6. Arenas, M.; Gorostiza, A.; Baquero, J.M.; Campoy, E.; Branco, C.; Rangel-Villalobos, H.; González-Martín, A. The Early Peopling of the Philippines Based on MtDNA. *Sci. Rep.* **2020**, *10*, 4901.
7. Ray, N.; Excoffier, L. A First Step towards Inferring Levels of Long-Distance Dispersal during Past Expansions. *Mol. Ecol. Resour.* **2010**, *10*, 902–914.
8. Wen, B.; Li, H.; Gao, S.; Mao, X.; Gao, Y.; Li, F.; Zhang, F.; He, Y.; Dong, Y.; Zhang, Y.; et al. Genetic Structure of Hmong-Mien Speaking Populations in East Asia as Revealed by MtDNA Lineages. *Mol. Biol. Evol.* **2004**, *22*, 725–734.
9. Tajima, A.; Sun, C.-S.; Pan, I.-H.; Ishida, T.; Saitou, N.; Horai, S. Mitochondrial DNA Polymorphisms in Nine Aboriginal Groups of Taiwan: Implications for the Population History of Aboriginal Taiwanese. *Hum. Genet.* **2003**, *113*, 24–33.
10. Trejaut, J.A.; Kivisild, T.; Loo, J.H.; Lee, C.L.; He, C.L.; Hsu, C.J.; Li, Z.Y.; Lin, M. Traces of Archaic Mitochondrial Lineages Persist in Austronesian-Speaking Formosan Populations. *PLOS Biol.* **2005**, *3*, e247.
11. Summerer, M.; Horst, J.; Erhart, G.; Weißensteiner, H.; Schönherr, S.; Pacher, D.; Forer, L.; Horst, D.; Manhart, A.; Horst, B.; et al. Large-Scale Mitochondrial DNA Analysis in Southeast Asia Reveals Evolutionary Effects of Cultural Isolation in the Multi-Ethnic Population of Myanmar. *BMC Evol. Biol.* **2014**, *14*, 17.

12. Pradutkanchana, S.; Ishida, T.; Kimura, R. Mitochondrial Diversity of the Sea Nomads of Thailand. *GenBank Access. Number—GU810073* **2010**.
13. Peng, M.-S.; Quang, H.H.; Dang, K.P.; Trieu, A.V.; Wang, H.-W.; Yao, Y.-G.; Kong, Q.-P.; Zhang, Y.-P. Tracing the Austronesian Footprint in Mainland Southeast Asia: A Perspective from Mitochondrial DNA. *Mol. Biol. Evol.* **2010**, *27*, 2417–2430.
14. Hanebuth, T.; Stattegger, K.; Grootes, P.M. Rapid Flooding of the Sunda Shelf: A Late-Glacial Sea-Level Record. *Science* **2000**, *288*, 1033–1035.
15. Soares, P.; Trejaut, J.A.; Loo, J.-H.; Hill, C.; Mormina, M.; Lee, C.-L.; Chen, Y.-M.; Hudjashov, G.; Forster, P.; Macaulay, V.; et al. Climate Change and Postglacial Human Dispersals in Southeast Asia. *Mol. Biol. Evol.* **2008**, *25*, 1209–1218.
16. Brandão, A.; Eng, K.K.; Rito, T.; Cavadas, B.; Bulbeck, D.; Gandini, F.; Pala, M.; Mormina, M.; Hudson, B.; White, J.; et al. Quantifying the Legacy of the Chinese Neolithic on the Maternal Genetic Heritage of Taiwan and Island Southeast Asia. *Hum. Genet.* **2016**, *135*, 363–376.
17. Hill, C.; Soares, P.; Mormina, M.; Macaulay, V.; Clarke, D.; Blumbach, P.B.; Vizuete-Forster, M.; Forster, P.; Bulbeck, D.; Oppenheimer, S.; et al. A Mitochondrial Stratigraphy for Island Southeast Asia. *Am. J. Hum. Genet.* **2007**, *80*, 29–43.
18. Macaulay, V.; Hill, C.; Achilli, A.; Rengo, C.; Clarke, D.; Meehan, W.; Blackburn, J.; Semino, O.; Scozzari, R.; Cruciani, F.; et al. Single, Rapid Coastal Settlement of Asia Revealed by Analysis of Complete Mitochondrial Genomes. *Science* **2005**, *308*, 1034 LP – 1036.
19. Hill, C.; Soares, P.; Mormina, M.; Macaulay, V.; Meehan, W.; Blackburn, J.; Clarke, D.; Raja, J.M.; Ismail, P.; Bulbeck, D.; et al. Phylogeography and Ethnogenesis of Aboriginal Southeast Asians. *Mol. Biol. Evol.* **2006**, *23*, 2480–2491.
20. Tommaseo-Ponzetta, M.; Attimonelli, M.; De Robertis, M.; Tanzariello, F.; Saccone, C. Mitochondrial DNA Variability of West New Guinea Populations. *Am. J. Phys. Anthropol.* **2002**, *117*, 49–67.
21. Gomes, S.M.; Bodner, M.; Souto, L.; Zimmermann, B.; Huber, G.; Strobl, C.; Röck, A.W.; Achilli, A.; Olivieri, A.; Torroni, A.; et al. Human Settlement History between Sunda and Sahul: A Focus on East Timor (Timor-Leste) and the Pleistocenic MtDNA Diversity. *BMC Genomics* **2015**, *16*, 70.
22. Hudjashov, G.; Kivisild, T.; Underhill, P.A.; Endicott, P.; Sanchez, J.J.; Lin, A.A.; Shen, P.; Oefner, P.; Renfrew, C.; Villems, R.; et al. Revealing the Prehistoric Settlement of

Australia by Y Chromosome and MtDNA Analysis. *Proc. Natl. Acad. Sci.* **2007**, *104*, 8726–8730.

23. Alves, I.; Arenas, M.; Currat, M.; Hanulova, A.S.; Sousa, V.C.; Ray, N.; Excoffier, L. Long-Distance Dispersal Shaped Patterns of Human Genetic Diversity in Eurasia. *Mol. Biol. Evol.* **2016**, *33*, 946–958.
24. Pimenta, J.; Lopes, A.M.; Comas, D.; Amorim, A.; Arenas, M. Evaluating the Neolithic Expansion at Both Shores of the Mediterranean Sea. *Mol. Biol. Evol.* **2017**, *34*, 3232–3242.
25. Oppenheimer, S. Out-of-Africa, the Peopling of Continents and Islands: Tracing Uniparental Gene Trees across the Map. *Philos. Trans. R. Soc. B Biol. Sci.* **2012**, *367*, 770–784.
26. Li, H.; Durbin, R. Inference of Human Population History from Individual Whole-Genome Sequences. *Nature* **2011**, *475*, 493–493.
27. Gravel, S.; Henn, B.M.; Gutenkunst, R.N.; Indap, A.R.; Marth, G.T.; Clark, A.G.; Yu, F.; Gibbs, R.A.; The 1000 Genomes Project; Bustamante, C.D. Demographic History and Rare Allele Sharing among Human Populations. *Proc. Natl. Acad. Sci.* **2011**, *108*, 11983.
28. Arenas, M.; François, O.; Currat, M.; Ray, N.; Excoffier, L. Influence of Admixture and Paleolithic Range Contractions on Current European Diversity Gradients. *Mol. Biol. Evol.* **2013**, *30*, 57–61.
29. Jinam, T.A.; Hong, L.-C.; Phipps, M.E.; Stoneking, M.; Ameen, M.; Edo, J.; HUGO Pan-Asian SNP Consortium; Saitou, N. Evolutionary History of Continental Southeast Asians: "Early Train" Hypothesis Based on Genetic Analysis of Mitochondrial and Autosomal DNA Data. *Mol. Biol. Evol.* **2012**, *29*, 3513–3527.
30. Soares, P.; Ermini, L.; Thomson, N.; Mormina, M.; Rito, T.; Röhl, A.; Salas, A.; Oppenheimer, S.; Macaulay, V.; Richards, M.B. Correcting for Purifying Selection: An Improved Human Mitochondrial Molecular Clock. *Am. J. Hum. Genet.* **2009**, *84*, 740–759.
31. Henn, B.M.; Gignoux, C.R.; Feldman, M.W.; Mountain, J.L. Characterizing the Time Dependency of Human Mitochondrial DNA Mutation Rate Estimates. *Mol. Biol. Evol.* **2009**, *26*, 217–230.
32. Madrigal, L.; Posthumously, L.C.; Melendez-Obando, M.; Villegas-Palma, R.; Barrantes, R.; Raventos, H.; Pereira, R.; Luiselli, D.; Pettener, D.; Barbujani, G. High

Mitochondrial Mutation Rates Estimated from Deep-Rooting Costa Rican Pedigrees. *Am. J. Phys. Anthropol.* **2012**, *148*, 327–333.
